# Interplay Between Notch and YAP/TAZ Pathways in the Regulation of Cell Fate During Embryo Development

**DOI:** 10.3389/fcell.2021.711531

**Published:** 2021-08-19

**Authors:** Carolyn Engel-Pizcueta, Cristina Pujades

**Affiliations:** Department of Experimental and Health Sciences, Universitat Pompeu Fabra, Barcelona, Spain

**Keywords:** YAP/TAZ pathway, Notch signaling pathway, cell fate, embryonic development, mechanical cues

## Abstract

Cells in growing tissues receive both biochemical and physical cues from their microenvironment. Growing evidence has shown that mechanical signals are fundamental regulators of cell behavior. However, how physical properties of the microenvironment are transduced into critical cell behaviors, such as proliferation, progenitor maintenance, or differentiation during development, is still poorly understood. The transcriptional co-activators YAP/TAZ shuttle between the cytoplasm and the nucleus in response to multiple inputs and have emerged as important regulators of tissue growth and regeneration. YAP/TAZ sense and transduce physical cues, such as those from the extracellular matrix or the actomyosin cytoskeleton, to regulate gene expression, thus allowing them to function as gatekeepers of progenitor behavior in several developmental contexts. The Notch pathway is a key signaling pathway that controls binary cell fate decisions through cell–cell communication in a context-dependent manner. Recent reports now suggest that the crosstalk between these two pathways is critical for maintaining the balance between progenitor maintenance and cell differentiation in different tissues. How this crosstalk integrates with morphogenesis and changes in tissue architecture during development is still an open question. Here, we discuss how progenitor cell proliferation, specification, and differentiation are coordinated with morphogenesis to construct a functional organ. We will pay special attention to the interplay between YAP/TAZ and Notch signaling pathways in determining cell fate decisions and discuss whether this represents a general mechanism of regulating cell fate during development. We will focus on research carried out in vertebrate embryos that demonstrate the important roles of mechanical cues in stem cell biology and discuss future challenges.

## Cell and Tissue Mechanics During Embryonic Development

How is a functional multiorgan system generated from a single, pluripotent cell? This fascinating question was already a major focus in D′Arcy Thompson’s book *On Growth and Form* (Thompson, 1917), in which he discussed the mechanisms by which organisms acquire their final sizes and shapes through growth. The understanding of how cell fate specification and proliferation are coordinated with tissue morphogenesis is crucial for unveiling the mechanisms underlying both normal and pathological tissue growth. Although classical developmental studies have been mainly focused on the role of biochemical signals, mechanical forces also play an important role in coordinating cell behavior with tissue morphogenesis (reviewed in Heer and Martin, 2017; Kumar et al., 2017; Labernadie and Trepat, 2018). Cells in developing tissues sense mechanical forces through cell–extracellular matrix (ECM) and cell–cell contacts, which are transmitted within and between cells through the cytoskeleton and adhesion molecules. Most of the current work in the mechanobiology field is devoted to the understanding of how forces drive the arrangement of cells in space such as cell intercalation, cell migration, or collective cell migration during self-organization and spreading of tissues. From those studies we know how forces produced by oriented cell division and growth, directed cell crawling or bending of cell sheets, integrate local cell shape changes (for reviews see Heisenberg and Bellaïche, 2013; Collinet and Lecuit, 2021). However, how such mechanical forces influence cell fate is still an open question. The molecular mechanisms driven by mechanical forces that control cell behaviors are partially understood. Nevertheless, we still do not have a comprehensive view of how the molecular mechanisms within cells are converted to mechanical forces during development. While the role of mechanical forces in cell fate can be studied in stem cell cultures, *in vitro* approaches do not provide insight into morphogenesis. Thus, understanding how mechanical signals control specific cell behaviors during morphogenesis is key to shedding light on how an organism is generated.

In this review, we will address the roles that mechanical cues have in binary cell fate decisions in different vertebrate developing tissues. Due to the increasing literature in the very last years and space limitations, we will cover a subset of representative studies in the field. Specifically, we will focus on the interplay between the highly conserved YAP/TAZ and Notch pathways. We will highlight the main findings of the recent research and discuss some of the unknowns in the field.

## The Usual Suspects: YAP/TAZ–TEAD as Sensors and Transducers of Mechanical Changes

### The Core of YAP/TAZ-TEAD

In the last few years, studies have started to disentangle how mechanical signals are interpreted by cells during morphogenesis, and how this results in specific cell behaviors. In this section, we will focus on the role of YAP/TAZ in this process.

The transcriptional co-activators Yes-associated protein (YAP) and its paralog TAZ (transcriptional co-activator with a PDZ binding domain; encoded by the *wwtr1* gene) are important regulators of tissue growth and regeneration (as reviewed in Hansen et al., 2015). YAP and TAZ regulation is best understood under the scope of the Hippo kinase cascade (Lei et al., 2008; Zhao et al., 2008). The Hippo pathway was initially identified through mosaic genetic screens for suppressors of tissue overgrowth in *Drosophila melanogaster* (Udan et al., 2003). Importantly, Hippo signaling cascade controls organ size and tissue homeostasis through the regulation of cell proliferation, apoptosis, and tissue regeneration (see review Zheng and Pan, 2019). Not surprisingly, deregulation of the pathway has been implicated in varieties of cancers and diseases (Plouffe et al., 2015). The core components of the Hippo pathway, the kinase Hippo (Hpo, or MST1 and MST2 in vertebrates), the kinase Warts (Wts, or LATS1 and LATS2 in vertebrates), and the effector Yorkie (Yki, or YAP and TAZ in vertebrates) are highly conserved from *Drosophila* to mammals (Hilman and Gat, 2011; Sebé-Pedrós et al., 2012). Despite the conservation of the core players, the upstream regulators of the pathway seem to be divergent (Hansen et al., 2015).

Activation of the Hippo kinase cascade results in the phosphorylation of Yki/YAP/TAZ, which inhibits their nuclear import. The upstream kinases of the cascade (Hippo or MST1/2) form a complex with the adaptor protein Salvador (SAV1 in vertebrates) that activates LATS1/2 kinases. LATS1/2 together with MATS/MOB1 phosphorylate and inactivate Yki (YAP and TAZ in vertebrates) by cytoplasmic retention and eventually ubiquitination and degradation (as reviewed in Panciera et al., 2017; Zheng and Pan, 2019). On the other hand, when Hippo is inactive, dephosphorylated YAP/TAZ can translocate into the nucleus and bind to the TEAD(1–4) transcription factors (Lei et al., 2008; Zhao et al., 2008). The YAP/TAZ–TEAD complex activates the expression of target genes that regulate cell proliferation, differentiation, and apoptosis (Dong et al., 2007; Lei et al., 2008; Zhao et al., 2008; Lian et al., 2010; Lee and Yonehara, 2012). Specifically, the YAP–TEAD complex controls gene transcription by mostly binding to distal enhancers (Galli et al., 2015). YAP/TAZ mainly act as co-activators but can also act as co-repressors together with TEAD factors (Beyer et al., 2013; Kim et al., 2015). Moreover, YAP/TAZ can bind to other transcription factors either with or without TEAD (as reviewed in Totaro et al., 2018b). Overall, although YAP/TAZ are the main known mediators of the Hippo pathway during development, YAP/TAZ-activity can be regulated by multiple microenvironmental cues beyond the Hippo pathway.

### YAP and TAZ as Mechanotransducers

In the last years, YAP/TAZ have emerged as sensors of mechanical forces (Dupont et al., 2011; Panciera et al., 2017). YAP/TAZ mechanotransduction can be triggered by cell density –either by cell–cell adhesion (see below), or reduced cell area (Aragona et al., 2013; Benham-Pyle et al., 2015)–, ECM rigidity (Dupont et al., 2011; Aragona et al., 2013; Elosegui-Artola et al., 2016), and shear stress (Nakajima et al., 2017). This regulation can be dependent or independently of the Hippo pathway. Within the cell density context, cell junction proteins regulate YAP/TAZ activity. For instance, Neurofibromatosis type 2 (NF2) works as a scaffold of the Hippo cascade components in cell–cell junctions. NF2 recruits LATS1/2 to the plasma membrane, enabling LATS1/2 activation by MTS1/2, which leads to inhibition of YAP/TAZ activity (Yin et al., 2013). Focal adhesions components, which contact the cell at the adjacent ECM, also regulate YAP/TAZ activity by modulating YAP subcellular location (Kim and Gumbiner, 2015; Elosegui-Artola et al., 2016). ECM stiffness and cell geometry control YAP/TAZ activity through small RhoGTPases triggering the actomyosin cytoskeleton tension in a Hippo independent manner (Dupont et al., 2011). Accordingly, F-actin inhibitor proteins mediate the spatial distribution of YAP/TAZ activity by mechanical forces along the tissue (Aragona et al., 2013). Forces exerted from the ECM can drive YAP/TAZ activity through different mechanisms. The Ras-related GTPase RAP2 transduces ECM stiffness into YAP/TAZ cellular responses through the activation of the Hippo pathway (Meng et al., 2018). The nuclear SWI/SNF complex inhibits YAP/TAZ as a response to mechanical signaling, in such a manner that to trigger YAP/TAZ activity, both YAP/TAZ nuclear accumulation and SWI/SNF inhibition are required (Chang et al., 2018). ECM forces can regulate the transport through the nuclear pores driving YAP nuclear import in a Hippo independent manner (Elosegui-Artola et al., 2017). Moreover, Piezo1, a mechanosensitive ion channel, can mediate the effects of substrate stiffness on YAP nuclear location (Pathak et al., 2014). Remarkably, YAP/TAZ are not only modulated by mechanical forces but can contribute to changes in actomyosin-mediated mechanical forces in cell culture and in developing tissues by regulating the expression of cytoskeletal and ECM genes (Porazinski et al., 2015; Lin et al., 2017; Nardone et al., 2017). Furthermore, biochemical cues also control YAP/TAZ activity (for recent reviews see Pocaterra et al., 2020; Heng et al., 2021). Extracellular cues can activate or inhibit YAP/TAZ activity through G-protein–coupled receptors (GPCRs). RhoGTPases mediate YAP/TAZ activation by GPCRs, modulating actomyosin cytoskeleton tension (Yu et al., 2012). Altogether, YAP/TAZ act as core integrators of chemical and mechanical cues in different biological contexts.

### YAP/TAZ in the Control of Cell Proliferation During Development

YAP/TAZ function as gatekeepers of progenitor cell behavior in several contexts during embryonic development. Specifically, YAP/TAZ have mainly been described as regulators of cell proliferation and tissue growth (Camargo et al., 2007; Lei et al., 2008; Ota and Sasaki, 2008). YAP/TAZ trigger proliferation of gastrointestinal mesenchymal progenitors (Cotton et al., 2017) and cranial neural crest cells (Wang et al., 2016). YAP by itself also controls the proliferation of cardiomyocytes (Heallen et al., 2011) and lung epithelial cells (Lin et al., 2017). Moreover, YAP–TEAD maintains inner ear progenitors through the activation of cell cycle and stemness genes (Gnedeva et al., 2020). In the same line, YAP/TAZ regulate cell proliferation in neural progenitors in the chick spinal cord by controlling their stemness properties through the activation of the cell cycle regulator *cyclinD1* and the inhibition of the neural differentiation marker *NeuroM* (Cao et al., 2008). YAP/TAZ–TEAD signaling also regulates the proliferation of neural progenitors in the mammalian embryonic brain (Han et al., 2015). YAP/TAZ drive the expansion of neural progenitors in the hippocampus downstream of NF2 (Lavado et al., 2013) and maintain neural progenitors in the developing cortex by activating the transcription of proliferation genes and preventing neural differentiation (Lavado et al., 2018). Along this, YAP also maintains the proliferative properties of basal progenitors in the developing ferret and human cortex (Kostic et al., 2019). Finally, YAP/TAZ–TEAD drives proliferation of neural progenitors in the zebrafish hindbrain boundaries downstream of actomyosin tension (Voltes et al., 2019). Although YAP/TAZ play essential roles in tissue growth during embryonic development, they are not required for normal physiology in most of adult tissues. Thus, while YAP/TAZ overexpression have a widely effect driving tissue hyper-proliferation and promoting tissue repair after injury, the deletion of YAP/TAZ in many adult contexts does not result in effects on tissue proliferation. Overall, YAP/TAZ play a crucial role in the self-renewal of progenitor cells during embryonic development.

## The Notch Pathway as the Key Regulator of Binary Cell Fate Decisions in Development

### Binary Cell Fate Decisions Through Cell–Cell Communication

The Notch signaling pathway is the main regulator of binary cell fate decisions during embryonic development (Artavanis-Tsakonas et al., 1999). Notch signaling operates through cell–cell communication, with one cell displaying the Notch receptor and its neighboring cell the Notch ligand. Nevertheless, Notch receptors and ligands can also form *cis* interactions that inhibit (de Celis and Bray, 1997; Micchelli et al., 1997) or activate (Nandagopal et al., 2019) Notch signaling in a cell-autonomous manner. The Notch pathway is highly conserved in metazoan species (Gazave et al., 2009). In mammals, there are four Notch receptors (Notch1–4) and five ligands: three Delta ligands (Dll1, Dll3, and Dll4) and two Jagged ligands (Jag1 and Jag2). Upon ligand binding (Delta or Jagged) in its extracellular domain, the Notch receptor undergoes several protease cleavages, thereby releasing the Notch Intracellular Domain (NICD), which then translocates into the nucleus. Once there, the NICD forms a complex with the transcription factor RBPJ (Recombination signal-Binding Protein for Ig Kappa J region) and recruits co-activators such as MAML (Mastermind-Like). The NICD–RBPJ complex activates transcription of the main effectors of the pathway, the transcriptional repressors genes *Enhancer of Split* (*Espl*) in *Drosophila* and *Hes/Her* in vertebrates. The Hes/Her transcription factors repress genes driving cell specification (e.g., proneural genes), cell differentiation, and cell cycle arrest (reviewed in Kageyama et al., 2007). Hes/Her can also repress – directly or indirectly – Notch ligand expression. Through this lateral inhibition mechanism, one cell is singled out from an equipotent field to acquire a specific fate, repressing this specific fate in the neighboring cells (reviewed in Henrique and Schweisguth, 2019).

However, the lateral inhibition paradigm should not only be viewed from a static perspective. Some of the Notch effectors, such as *Hes1* and *Hes7*, show an oscillatory expression by a negative autoregulatory loop in different developing tissues (Hirata et al., 2002; Bessho et al., 2003; Lahmann et al., 2019; Seymour et al., 2020), resulting in the oscillation of their targets (Masamizu et al., 2006; Shimojo et al., 2008; Lahmann et al., 2019). The oscillation of *Hes* genes and their targets keep progenitors in an undifferentiated and proliferative state, whereas the sustained expression of one of the target genes drives cell specification (Shimojo et al., 2008; Lahmann et al., 2019). Overall, *Hes* oscillations constitute a crucial mechanism in the control of binary cell fate decisions during embryonic development.

Moreover, Notch not only controls cell fate through lateral inhibition, but also through lateral induction (de Celis and Bray, 1997). Lateral induction consists of a positive feedback loop in which Notch signaling activates the expression of the Notch ligand, thereby activating Notch signaling in the adjacent cell. Subsequently, both interacting cells acquire the same fate. For instance, Jag1–Notch signaling through lateral induction drives prosensory fate in the developing inner ear (Hartman et al., 2010) and vascular smooth muscle fate in neural crest cells (Manderfield et al., 2012). This highlights another level of complexity in the control of cell fate decisions by Notch signaling in the developing embryo.

### Context Dependency of Notch Control of Cell Fate Decisions

The role of the Notch pathway during development is highly context dependent (Bray, 2016). In vertebrates, different combinations and spatiotemporal expression of Notch receptors and ligands account for part of this context dependency. For example, the control of neuronal fates in the zebrafish spinal cord relies on different combinations of Notch ligands and receptors (Okigawa et al., 2014). Different ligands can also trigger distinct responses through the same Notch receptor. For instance, Jagged and Delta ligands drive different outcomes in the control of cell fate decisions during inner ear development and angiogenesis (Benedito et al., 2009; Petrovic et al., 2014). Likewise, Dll1 and Dll4 induce different Notch activation dynamics, driving different gene programs and cell fates (Nandagopal et al., 2018). Additionally, Fringe glycotransferases modify the affinity between Notch receptors and ligands (Panin et al., 1997), providing an extra regulatory layer. Epigenetic mechanisms may explain part of the Notch context dependency. For example, epigenetic modifications in the regulatory regions of Notch targets regulate cell fate decisions in olfactory and cortical neurogenesis (Endo et al., 2011; Tiberi et al., 2012). Moreover, the NICD–RBPJ complex can interact with other transcription factors (reviewed in Bray, 2016). Hence, the interplay between Notch signaling and other pathways is relevant for the diversity of Notch responses. Cell geometry is also crucial for Notch regulation of cell fate during development (Shaya et al., 2017). All this complexity raises the question of how the interactions between Notch signaling, the microenvironment and other signaling pathways contribute to Notch pleiotropic effects during development.

## Mechanosensing in the Control of Cell Fate Decisions

Notch controls binary cell fate decisions during morphogenesis, while YAP/TAZ transduce physical properties of the microenvironment into critical cell decisions. In this chapter, we will discuss the different described biological roles of Notch and YAP/TAZ, and their interplay in the control of binary cell fates in several contexts.

YAP/TAZ regulate cell fate in response to mechanical signals in different tissues. In the preimplantation embryo, YAP/TAZ–TEAD promote the specification of the trophectoderm fate (Nishioka et al., 2009). In the nervous system, YAP controls neocortical astrocyte (Huang et al., 2016) and retinal pigment epithelium fate (Miesfeld et al., 2015; Kim et al., 2016). In the kidney, YAP drives nephron differentiation downstream of the small RhoGTPase Cdc42 (Reginensi et al., 2013). YAP–TEAD also regulates the program of airway epithelial progenitor specification (Mahoney et al., 2014) and the hematopoietic stem cell fate in response to cyclic stretch (Lundin et al., 2020). Furthermore, YAP/TAZ inhibit smooth muscle cell differentiation in the developing gut (Cotton et al., 2017) and control the formation of the signaling center, the enamel knot, during tooth development (Li et al., 2016). TAZ (without YAP) also controls cell specification such as micropyle precursor cell fate during zebrafish oogenesis (Dingare et al., 2018). Specifically, TAZ activity singles out this micropyle precursor cell through a lateral inhibition mechanism based on differential cell growth, generating pushing forces that exclude nuclear TAZ in the neighboring cells (Xia et al., 2019). This mechanism challenges the classic paradigm of lateral inhibition being an exclusively Notch–Delta mechanism to singularize cells from an equipotent cell field. Altogether, YAP/TAZ act as integrators of mechanical inputs, regulating the balance between progenitor and differentiated cells in different developmental contexts.

Moreover, Notch has also been proposed to be a sensor of the microenvironment (reviewed in Lloyd-Lewis et al., 2019; Stassen et al., 2020). At the molecular level, pulling forces from the sending cell can activate Notch1 signaling (Gordon et al., 2015; Chowdhury et al., 2016). The main examples of this have been described in the vascular system. In adult arteries, Notch1 works as a mechanosensor downstream of shear stress, controlling arterial identity and proliferation in endothelial cells (Mack et al., 2017). Similarly, the Notch3–Jag1 complex senses mechanical cues, potentially regulating the behavior of vascular smooth muscle cells (Loerakker et al., 2018). However, Notch1 is necessary but not sufficient to transduce the shear stress generated by the onset of blood flow in mouse embryos (Jahnsen et al., 2015). Hence, Notch is involved in the regulation of these processes in collaboration with other mechanosensors. Along this line, endothelial Piezo1 triggers a Notch1 response to mechanical signals by activating the metalloprotease responsible for the Notch receptor intracellular cleavage (Caolo et al., 2020). Overall, these studies point to Notch as a putative sensor and transducer of mechanical forces. Whether Notch controls cell fate during development downstream of mechanical forces alone or in collaboration with other mechanosensors remains an open question.

**FIGURE 1:**
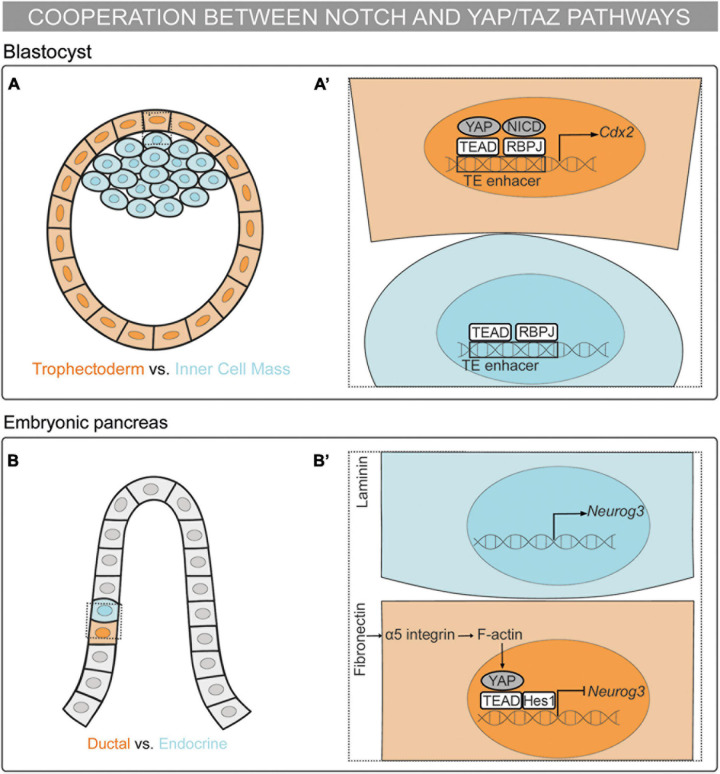
Mechanosensing in the control of cell fate decisions: the cooperation between the Notch and YAP/TAZ pathways. **(A,A’)** Schematic representation of a mouse blastocyst. Trophectoderm (TE) cells are depicted in orange and Inner Cell Mass (ICM) cells in blue. **(A’)** Magnification of the framed region in **(A)**. RBPJ and YAP–TEAD bind to the *Cdx2* TE-enhancer and drive the TE fate (Rayon et al., 2014; Watanabe et al., 2017; Menchero et al., 2019). **(B,B’)** Schematic representation of a mouse embryonic pancreas. Cells fated to the ductal linage are colored in orange and cells fated to the endocrine lineage in blue. Multipotent pancreatic progenitors are depicted in gray. **(B’)** Magnification of the framed region in **(B)**. The pancreatic progenitor cells sensing fibronectin activate the α5-integrin–F-actin-YAP axis. YAP–TEAD complex activates *Hes1* expression. Further, Hes1 binds to the YAP–TEAD complex repressing *Neurog3* expression and committing the progenitor cell to the ductal lineage (Mamidi et al., 2018).

**FIGURE 2:**
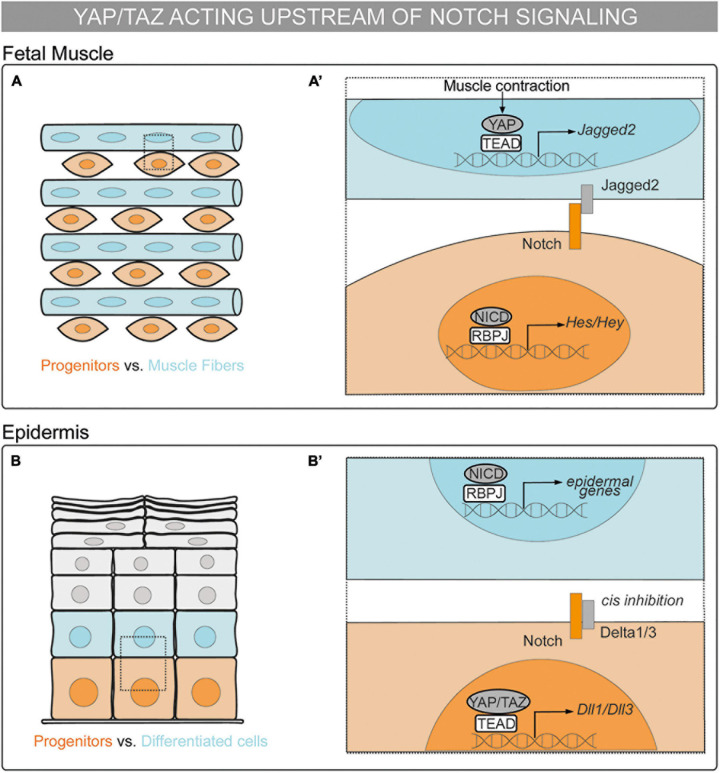
Mechanosensing in the control of cell fate decisions: the YAP/TAZ pathway acting upstream of Notch signaling in cell-autonomous vs. non–cell-autonomous mechanisms. **(A,A’)** Schematic representation of a chick fetal muscle. Muscle progenitors are depicted in orange and muscle fibers in blue. **(A’)** Magnification of the framed region in **(A)**. Upon muscle contraction, YAP translocates to the nucleus, binds to TEAD, and activates the transcription of *Jag2*. Jagged2 ligand activates Notch receptor in the neighboring cell and maintains the muscle progenitor in a non–cell-autonomous manner (Esteves de Lima et al., 2016). **(B,B’)** Schematic representation of a mammalian epidermis. Epidermal progenitors are depicted in orange and cells committing to the epidermal fate in blue. Differentiated epidermal cells are depicted in gray. **(B’)** Magnification of the framed region in **(B)**. YAP/TAZ–TEAD drive the expression of *Dll1* and *Dll3* that *cis* inhibit Notch signaling in basal progenitors, preventing epidermal differentiation in a cell-autonomous manner (Totaro et al., 2017).

**FIGURE 3:**
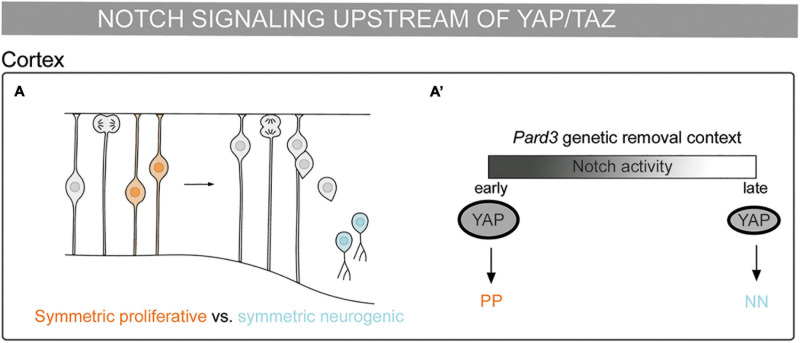
Mechanosensing in the control of cell fate decisions: Notch pathway being upstream of YAP/TAZ. **(A)** Schematic representation of a mouse embryonic cortex. Progenitor cells from a symmetric proliferative division are depicted in orange and two neurons deriving from a symmetric neurogenic division are depicted in blue. **(A’)** Scheme of cell division modes in the *Pard3* genetic deletion context. At the early neurogenic phase, Notch activity increases YAP expression and promotes symmetric proliferative divisions (orange). At late neurogenic phase, the decrease of Notch activity results in lower YAP expression and the promotion of symmetric neurogenic divisions (blue) (Liu et al., 2018).

In recent years, the interplay between YAP/TAZ and Notch has been proposed to regulate a wide range of biological processes. In the next section, we will cover the crosstalk between YAP/TAZ–TEAD and Notch signaling as a key link between mechanical signals and binary cell fate decisions during embryonic development. Specifically, we will describe the different following modes of action proposed to date: (i) the cooperation between the Notch and YAP/TAZ pathways (Figure 1); (ii) YAP/TAZ acting upstream of Notch signaling (Figure 2); and (iii) Notch signaling acting upstream of YAP/TAZ (Figure 3).

### Cooperation Between the Notch and YAP/TAZ Pathways in Binary Cell Fate Decisions

The first binary cell fate decision in the mammalian embryo occurs during the transition from morula to blastocyst, with the decision made between becoming trophectoderm (TE) or inner cell mass (ICM). YAP/TAZ–TEAD drive the specification of the TE fate downstream of cell polarity through Hippo-dependent and independent mechanisms (Nishioka et al., 2009; Cockburn et al., 2013; Hirate et al., 2013; Leung and Zernicka-Goetz, 2013; Lorthongpanich et al., 2013). In the blastocyst, Notch is specifically activated in the outer cells, which will give rise to the TE. Notch and YAP–TEAD activate the expression of the TE specification gene, *Cdx2*, by binding to its TE-specific enhancer (Rayon et al., 2014). Moreover, the helicase-like protein Strawberry Notch1 (Sbno1) interacts with the YAP–TEAD and NICD–RBPJ complexes, operating as an integrator of these complexes in the activation of the TE-enhancer of *Cdx2* (Watanabe et al., 2017). This activation is not redundant since Notch regulates the onset of *Cdx2* expression whereas YAP–TEAD maintains *Cdx2* expression. Thus, through this mechanism, YAP–TEAD and Notch cooperate in the specification of the TE in a parallel and independent manner (Menchero et al., 2019). *In vitro* stretching of embryonic stem cells results in the activation of the TE enhancer of *Cdx2* in the presence of YAP, TEAD, and Notch (Watanabe et al., 2017). Therefore, mechanical forces are upstream of the Notch and YAP–TEAD synergic mechanism in the binary cell fate decision between TE or ICM (Figures 1A,A’).

The embryonic pancreas contains multipotent progenitors organized in tubular epithelial structures formed by a tip and a trunk domain. Bipotent pancreatic progenitors residing in the trunk domain give rise to the ductal and endocrine cells. The YAP–TEAD complex acts as a main regulator for the maintenance of human pancreatic progenitors by activating several targets, including *Hes1* (Cebola et al., 2015). Further, YAP–TEAD forms a transcriptional complex with Hes1 that represses the expression of the endocrine specification gene, *Neurog3*. Importantly, mechanical signals regulate this cell fate decision both *in vivo* and *in vitro*: cell confinement drives endocrine specification whereas cell spreading triggers ductal specification. Accordingly, different ECM compositions define the lineage commitment of the mouse and human pancreatic progenitors. Pancreatic progenitors sensing fibronectin activate the YAP–TEAD–Hes1 complex through the α5-integrin–F-actin axis, thereby repressing the endocrine cell fate. On the other hand, progenitors sensing laminin reduce the activation of the α5-integrin–YAP–Hes1 axis, committing to the endocrine fate (Mamidi et al., 2018). In this scenario, YAP–TEAD is upstream of Notch signaling meanwhile cooperating with Hes1 in the repression of *Neurog3* expression to maintain pancreatic progenitors, which leads to the default commitment to the ductal lineage (Figures 1B,B’).

YAP/TAZ forms a common transcriptional complex with the NICD in the control of smooth muscle differentiation from neural crest cells (Manderfield et al., 2015). Notch signaling plays a critical role in the differentiation of cardiac neural crest cells into smooth muscle cells through lateral induction (High et al., 2007). Firstly, the vascular endothelium displays a Jag1 ligand, which activates Notch signaling in the neighboring mesenchyme. Subsequently, Notch activates the smooth muscle differentiation program and *Jag1* transcription, generating a positive feedback loop that controls smooth muscle differentiation (Manderfield et al., 2012). Specific deletion of YAP and TAZ in neural crest cells impairs smooth muscle differentiation. YAP/TAZ deletion decreases *Jag1* and NICD expression in the mesenchyme, while NICD expression remains intact in endothelial cells. In this context, YAP physically interacts in a TEAD-independent manner with the NICD–RBPJ complex, activating *Jag1* enhancer and the *Hes1* promoter *in vivo* and *in vitro* (Manderfield et al., 2015). This common YAP–NICD–RBPJ transcriptional complex contrasts with the parallel cooperation of YAP and NICD in the trophectoderm (Rayon et al., 2014; Watanabe et al., 2017; Menchero et al., 2019). In brief, Notch and YAP cooperate to activate the transcription of Notch targets controlling smooth muscle fate. Noteworthy, the vascular tissue is highly exposed to mechanical forces during development. Therefore, mechanical forces could be controlling smooth muscle fate through the regulation of YAP/TAZ and Notch.

### The YAP/TAZ Pathway Acting Upstream of Notch Signaling in Cell-Autonomous vs. Non–cell-Autonomous Mechanisms

YAP/TAZ can act upstream of Notch signaling by activating Notch receptors. Notch participates in the binary decision between cholangiocytes and hepatocytes (Kodama et al., 2004; Zong et al., 2009). YAP controls the proliferation of hepatocytes and hepatic progenitors downstream of the Hippo pathway in the adult liver (Camargo et al., 2007; Zhou et al., 2009; Lee et al., 2010; Lu et al., 2010). In this context, YAP promotes the biliary cell fate downstream of NF2 regulation (Zhang et al., 2010). NF2 deficiency increases biliary precursors and cholangiocytes proliferation during development. Remarkably, Notch2 deficiency rescues this phenotype. In the absence of NF2, YAP activates *Notch2* expression controlling biliary specification and cholangiocytes proliferation (Wu et al., 2017). Thus, YAP controls biliary cell fate and proliferation through the activation of the *Notch2* receptor during intrahepatic bile duct development, as previously described in adult hepatocytes (Yimlamai et al., 2014). In the adult liver, YAP can also act upstream of Notch pathway by activating the expression of *Jag1* (Tschaharganeh et al., 2013). In contrast, YAP/TAZ and Notch have been proposed to promote biliary cell fate through parallel mechanisms during development (Lee et al., 2016). Finally, YAP/TAZ and Notch control the binary decision between cholangiocytes and hepatocytes downstream of mechanical forces in the adult liver (Pocaterra et al., 2019, 2021). Whether this binary cell fate decision is controlled by YAP/TAZ–Notch downstream of mechanical forces during embryonic development is still unsolved.

On the other hand, YAP can inhibit Notch activity in a cell-autonomous manner, as it occurs during angiogenesis and somitogenesis. During embryonic angiogenesis, Notch controls the cell fate decision of tip vs. stalk cell. In tip cells, the Notch ligand Dll4 triggers the formation of new sprouts and activates Notch in neighboring stalk cells, leading to tip fate suppression. Notch in stalk cells can also activate *Dll4* expression, thus triggering Notch activation in tip cells (Caolo et al., 2010). This mechanism maintains arterial identity while regulating the formation of new branches. YAP/TAZ are activated in tip cells through the activation of the GPCRs, LPA4 and LPA6, mediated by actomyosin tension. YAP/TAZ control sprouting angiogenesis through the blockage of βcatenin–NICD mediated endothelial *Dll4* expression. Altogether, YAP/TAZ inhibits *Dll4* expression in tip cells in a cell-autonomous and TEAD-independent manner to control sprouting angiogenesis (Yasuda et al., 2019). In other words, as in smooth muscle differentiation, YAP/TAZ acts in a TEAD-independent manner in the regulation of Notch ligand expression. The second case is observed in the presomitic mesoderm (PSM) for the genetic synchronous oscillations of the Notch target *Hes7* that precede the formation of somites, known as the segmentation clock. The segmentation clock has been proposed to be an excitable system (Hubaud et al., 2017). In this model, YAP activation provides an excitability threshold and Notch acts as the stimulus triggering the oscillations once it exceeds the threshold. Therefore, the collaboration of YAP and Notch is required for triggering and maintaining PSM oscillations. Importantly, YAP activation in the PSM cells is controlled by mechanical cues, such as cell density. In this scenario, Notch controls the decision between the quiescent and oscillatory state in PSM cells downstream of YAP activation by mechanical cues (Hubaud et al., 2017). In both contexts, YAP inhibits Notch signaling to control cell behavior in a cell-autonomous manner downstream of mechanical cues.

YAP can also be upstream Notch in a non–cell-autonomous manner. During fetal myogenesis, Notch controls the binary cell fate decision between muscle progenitors and differentiated muscle cells (Vasyutina et al., 2007). In the chick embryonic limb, nuclear YAP is expressed in differentiated muscle cells and in a subpopulation of muscle progenitors. YAP controls the maintenance but not proliferation of muscle progenitors downstream of muscle contraction. In muscle fibers, YAP–TEAD binds to the *Jag2* promoter and activates *Jag2* expression. Consistently, *Jag2* expression in muscle fibers as well as Notch in muscle progenitors decrease upon muscle immobilization (Esteves de Lima et al., 2016). Overall, YAP–TEAD drives *Jag2* expression in muscle fibers upon muscle contraction, as a result, Jag2 activates Notch in neighboring cells, maintaining the muscle progenitor cell pool in a non–cell-autonomous manner (Figures 2A,A’).

The YAP/TAZ–TEAD and Notch pathways do not always have synergic functions. In the epidermis, basal progenitors specify from the basement membrane to the tissue surface. Notch signaling participates in the cell decision between basal progenitor and the epidermal fate. The NICD–RBPJ complex drives the expression of epidermal differentiation genes (Blanpain et al., 2006). Low cell density or high ECM rigidity trigger YAP/TAZ–TEAD activation in basal progenitors leading to progenitor maintenance through the inhibition of Notch signaling, as shown both *in vitro* and *in vivo* (Totaro et al., 2017). This process is cell–cell contact independent. In basal progenitors, the YAP/TAZ–TEAD complex activates the transcription of *Dll1* and *Dll3*; thereafter, *cis* interactions of Dll1 and Dll3 with Notch receptors can block Notch activation, thus, preventing epidermal differentiation. Altogether, YAP/TAZ–TEAD controls epidermal fate decisions by inhibiting Notch signaling downstream of mechanical signals in a cell-autonomous manner (Figures 2B,B’).

### Notch Pathway Being Upstream of YAP/TAZ

Notch can be upstream of YAP/TAZ during embryonic brain development. Asymmetric divisions play a role in the balance between cell proliferation and differentiation. Radial glial progenitors (RGPs) divide asymmetrically to give rise to a neuron and another RGP. The polarity gene *Pard3* is highly expressed in the apical cell surface and regulates asymmetric divisions in RGPs in the mammalian cortex (Costa et al., 2008). Pard3 removal by genetic depletion in mice leads to temporally distinct changes in RGP mitotic behavior: at the early neurogenic phase, it results in increased YAP expression and promotes symmetric proliferative divisions, while at late neurogenic phase, it results in decreased YAP expression and promotes RGP symmetric differentiation divisions. Notch expression decreases during cortical development, coinciding with the RGPs behavioral switch. Accordingly, Notch promotes high YAP levels in the nucleus upon *Pard3* removal (Liu et al., 2018). This activation could be through the binding of the NICD–RBPJ complex to the YAP promoter, as described in neural stem cells (Li et al., 2012). Thus, the interplay of Pard3 with Notch and YAP/TAZ could explain the potential role that mechanical signals play in this process. Altogether, the Notch pathway upstream of YAP/TAZ controls the division cell mode and, therefore, cell fate in the developing cortex (Figures 3 A,A’).

## The Link Between Morphogenetic Changes and Cell Fate

Mechanical forces can control cell fate decisions. Seminal studies have widely demonstrated how mechanical signals influence the lineage commitment of multipotent stem cells (Sordella et al., 2003; McBeath et al., 2004; Hong et al., 2005; Engler et al., 2006; Kilian et al., 2010; Dupont et al., 2011; Tang et al., 2013). However, an important question that remains is how the morphogenetic tissue changes are intertwined with cell fate decisions during embryonic development. For this, several challenges need to be addressed, such as understanding the modulation of forces in embryos *in vivo* and how they result in distinct cell fates upon morphogenesis. Thus, the advancement in new techniques to study and manipulate mechanical forces in complex 3D structures *in vivo* will be crucial to elucidate how tissue morphogenesis and cell fate decisions are coupled. In the meantime, the best approach is to combine *in vitro* approaches, which allow mechanical forces to be precisely manipulated, with *in vivo* studies, which provide the whole tissue context. In this way, we can gain a better understanding of the interactions between the different cell types and their environment as well as of the mechanisms operating downstream of mechanical signals.

Interplay between Notch and YAP/TAZ can mediate the role of mechanical forces in binary cell fate decisions (see section “Mechanosensing in the Control of Cell Fate Decisions”), and it plays an important role in different pathologies, adult homeostasis, and regeneration (Totaro et al., 2018a). However, the role of TAZ has not been assessed in many developmental contexts, and in most of the cases the main functions have been associated to YAP. YAP and TAZ can act both redundantly, but also in a specific manner (Morin-Kensicki et al., 2006; Hossain et al., 2007; Makita et al., 2008; Reginensi et al., 2013; Talwar et al., 2021), depending on the biological process. An open question is whether YAP and TAZ act together in systems where the robustness needs to be maintained, or whether they have specific attributed roles. Along this line, future studies are needed as well to uncover whether TAZ can also form transcriptional complexes with NICD–RBPJ or HES1, or whether this is a specific YAP property. Remarkably, the interplay between Notch and YAP/TAZ results in cooperation in most developmental scenarios. This cooperation arises as a relevant mechanism to explain the missing link between mechanical cues and cell fate decisions in developing tissues.

Cell specification and proliferation are intertwined processes. YAP/TAZ are regulators of progenitor cell proliferation downstream of mechanical signals (Hansen et al., 2015), while Notch is known as the main regulator of binary cell fate decisions (Artavanis-Tsakonas et al., 1999). How do both pathways coordinate cell proliferation and specification during morphogenesis? Is the interplay between YAP/TAZ and Notch a common mechanism of most developing tissues? There are several speculative scenarios. Notch could operate first to establish a given cell fate. Then, this fate could be maintained by YAP/TAZ and the cell population would expand. On the other hand, mechanical signals could trigger YAP/TAZ activity to promote the maintenance and proliferation of the given cell population, until Notch activates the transition of cells toward specification and differentiation. In this case, YAP/TAZ may either activate or inhibit Notch signaling to start this transition. In both scenarios, Notch and YAP/TAZ can cooperate to either repress or drive a given cell fate. However, Notch and YAP/TAZ can control the fate and proliferation of the same cell population by different and parallel mechanisms. This could explain how YAP/TAZ can control cell fate independently of Notch signaling and how Notch can act as a mechanosensor independently of YAP/TAZ. Therefore, the cooperative role of Notch and YAP/TAZ downstream of mechanical signals could shed light into the coordination between cell specification and proliferation. Moreover, the role of other players in the interplay between Notch and YAP/TAZ illustrates another layer of complexity in their regulation and highlights their crucial role in the integration of extrinsic and intrinsic inputs. Altogether, Notch and YAP/TAZ interplay allows a better understanding of the pleiotropic effects of both pathways during development. Thus, Notch and YAP/TAZ interplay could be seen as a core mechanism linking mechanical cues and binary cell fate decisions. Nevertheless, their independent functions and the interplay with other signaling pathways points to a model with higher complexity. Further studies need to be conducted to uncover the regulation and roles of Notch and YAP/TAZ during vertebrate development.

## Author Contributions

CE-P and CP contributed to the concept, design, and writing of the review. Both authors contributed to the article and approved the submitted version.

## Conflict of Interest

The authors declare that the research was conducted in the absence of any commercial or financial relationships that could be construed as a potential conflict of interest.

## Publisher’s Note

All claims expressed in this article are solely those of the authors and do not necessarily represent those of their affiliated organizations, or those of the publisher, the editors and the reviewers. Any product that may be evaluated in this article, or claim that may be made by its manufacturer, is not guaranteed or endorsed by the publisher.
